# The Role of Hypoxia-Inducible Factor-1**α**, Glucose Transporter-1, (GLUT-1) and Carbon Anhydrase IX in Endometrial Cancer Patients

**DOI:** 10.1155/2014/616850

**Published:** 2014-03-12

**Authors:** Pawel Sadlecki, Magdalena Bodnar, Marek Grabiec, Andrzej Marszalek, Pawel Walentowicz, Alina Sokup, Jolanta Zegarska, Małgorzata Walentowicz-Sadlecka

**Affiliations:** ^1^Department of Obstetrics and Gynecology, The Ludwik Rydygier Collegium Medicum in Bydgoszcz, Nicolaus Copernicus University of Torun, Ulica Ujejskiego 75, 85-168 Bydgoszcz, Poland; ^2^Department of Clinical Pathology, The Ludwik Rydygier Collegium Medicum in Bydgoszcz, Nicolaus Copernicus University of Torun, 87-100 Torun, Poland; ^3^Department of Gastroenterology, Angiology and Internal Diseases, The Ludwik Rydygier Collegium Medicum in Bydgoszcz, Nicolaus Copernicus University of Torun, 87-100 Torun, Poland

## Abstract

Hypoxia-inducible factor-1*α* (HIF-1*α*), glucose transporter-1 (GLUT-1), and carbon anhydrase IX (CAIX) are important molecules that allow adaptation to hypoxic environments. The aim of our study was to investigate the correlation between HIF-1*α*, GLUT-1, and CAIX protein level with the clinicopathological features of endometrial cancer patients. *Materials and Methods*. 92 endometrial cancer patients, aged 37–84, were enrolled to our study. In all patients clinical stage, histologic grade, myometrial invasion, lymph node, and distant metastases were determined. Moreover, the survival time was assessed. Immunohistochemical analyses were performed on archive formalin fixed paraffin embedded tissue sections. *Results*. High significant differences (*P* = 0.0115) were reported between HIF-1*α* expression and the histologic subtype of cancer. Higher HIF-1*α* expression was associated with the higher risk of recurrence (*P* = 0.0434). The results of GLUT-1 and CAIX expression did not reveal any significant differences between the proteins expression in the primary tumor and the clinicopathological features. *Conclusion*. The important role of HIF-1*α* in the group of patients with the high risk of recurrence and the negative histologic subtype of the tumor suggest that the expression of this factor might be useful in the panel of accessory pathomorphological tests and could be helpful in establishing more accurate prognosis in endometrial cancer patients.

## 1. Introduction

Endometrial cancer is the most frequent female genital malignancy in highly developed countries, with a life time risk of its development amounting to 2-3% [1]. The aim of current ongoing studies of endometrial cancer patients is to identify new factors found in tumor tissue or blood serum that could be used in order to predict prognosis, define optimal therapeutic protocol, and estimate the risk of recurrence.

Natural course of endometrial cancer is slow and the disease is characterized by rather good prognosis. Early onset of clinical symptoms enables us to set the diagnosis at the early stage of the disease. The 5-year overall survival (OS) rate of women with endometrial cancer is high, counting more than 80% for all stages and more than 90% for stage I [2]. Endometrial cancer is successfully treated with surgery and/or radiotherapy [3]. However, for patients with advanced or recurrent disease, only limited treatment options are available. There is a group of patients with a poor prognosis, who will benefit from more aggressive treatment. This group will need adjuvant chemo- or radiotherapy. It is of great interest to learn more about the important risk factors predictive of recurrence and/or death.

The recognized so far poor prognostic factors for endometrial cancer are advanced FIGO stage, a nonendometrioid histological subtype, high grade (G3), deep invasion of myometrium (>50%), presence of lymph node metastases, cervical involvement, and lymphovascular space invasion (LVSI) [2]. All risk factors mentioned above are identified after extensive surgical procedure and detailed pathologic report.

Even though our knowledge about tumor cells have improved a lot throughout recent years, the precise mechanisms that rule the process of tumor progression and metastases formation remain unknown. Hypoxia plays a vital role in carcinogenesis. Metabolic reprogramming and changes in gene expression are necessary for adaptation to decreased O2 availability in the tumor microenvironment. Hypoxia-inducible factors (HIFs) are oxygen-sensitive transcription factors that allow adaptation to hypoxic environments. HIFs are important mediators of the cellular response to stress e.g. metabolic, hypoxic, or inflammatory. Metabolic changes occur during tumorigenesis that are, in part, under hypoxia and HIF regulation. Additionally, inflammatory signaling and infiltration secondary to hypoxia is clear drivers of tumor progression [4]. However, despite the well-documented role of hypoxia in tumor microenvironment, its significance in endometrial cancer is not completely understood. HIF-1*α* is a key regulator of oxygen homeostasis in nearly all nuclear cells of mammals [4, 5]. Immunohistochemical studies revealed that many cancers are characterized by overexpression of HIF-1*α* as compared to normal tissues [6]. Adaptation to changing levels of cellular oxygen is determined mostly by the alpha subunit of HIF-1 (HIF-1*α*). Under hypoxemic conditions, the active factor HIF-1*α* is involved in the regulation of glucose metabolism, pH, angiogenesis, cellular differentiation, migration, and formation of metastases [7–12]. The metabolism of glucose in tumor microenvironment is changed from oxygen mitochondrial process to glycolysis (the Warburg effect) [13]. HIF-1*α* regulates the activity of glucose transporters (GLUTs), GLUT1 and GLUT3, that are responsible for glucose uptake [14–16]. Expression of GLUT1 increases under hypoxemic conditions what induces a shift in glucose metabolism towards glycolysis. The expression of GLUT-1 was revealed to be regulated by hypoxia in a HIF-1-dependent manner [17]. Carbon anhydrase IX (CAIX) is another factor associated with the activity of HIF-1*α* [18]. The effect of CAIX on tumor microenvironment is characterized by the regulation of pH. The overexpression of CAIX was observed in many cancer tissues but not in normal tissues [19].

The aim of this study was to verify the usefulness of HIF-1*α*, GLUT-1, and CAIX, determined immunohistochemically in primary tumor and analyzed together with other clinical parameters, in predicting prognosis and planning tailored treatment of endometrial cancer patients. The detailed objectives included determining expressions of these factors in primary tumor, analysis of their relationships with other clinicopathological characteristics of the tumor, verification of the usefulness of selected immunohistochemically determined proteins as predictors of unfavorable clinicopathological parameters in endometrial cancer patients, and analysis of relationship between expression of the studied proteins and 5-year survival rate.

## 2. Materials and Methods

### 2.1. Patients

92 endometrial cancer patients, aged 37–84 (mean 65.1 ± 9.5), were enrolled to our study between January 2000 and December 2007. After diagnosis of endometrial cancer based on specimens obtained from curettage, all patients underwent total abdominal hysterectomy, with bilateral salpingoophorectomy and pelvic lymph node dissection performed by experienced gynecological oncologists at Department of Oncologic Gynecology of LudwikRydygier Collegium Medicum in Bydgoszcz, Nicolaus Copernicus University. Clinical stage was assessed based on surgical specimens evaluation performed by two independent experienced pathologists according to International Federation of Gynecology and Obstetrics (FIGO) 2009 system. The study group included 27 patients with stage IA, 18 with stage IB, 14 with II, 10 women with stage IIIA, 17 with IIIC, and 6 with IV. Histological grade was assessed according to WHO classification. Histological grade 1 (G1) was noted in 7 patients, G2 in 66, and G3 in 19 women. Deep myometrial invasion (>50%) was observed in 36 patients, lymph node metastases in 23 women, distant metastases in 6, cervical involvement in 38, and adnexal involvement in 11 patients. Baseline characteristics of the study participants are enclosed in Table 1.

According to presently used risk factors, all patients were divided into three groups: low risk: FIGO IA, G1 or G2, and Bokhman type I (endometrioid); intermediate risk: IA G3, IB G1 or G2, and Bokhman type I (endometrioid); high risk: all patients in type II (nonendometrioid), IB G3, FIGO II, and higher. Patients from low risk group did not receive any further treatment after surgery, women from intermediate risk group received brachytherapy (VBT) 5 weeks after surgery, and patients from high risk group underwent teleradiotherapy and VBT. Adjuvant chemotherapy was administered to ten patients with nonendometrioid histopathological subtype (chemotherapy consisted of carboplatin and paclitaxel).

In all cases overall survival was determined (in months). Only cases with proven death related to cancer were analyzed. The follow-up time was 60–80 months.

The Ethical Committee at the Ludwik Rydygier Collegium Medicum, Nicolaus Copernicus University of Torun, approved this study protocol (decision number KB 332/2007). All participants have provided and signed the informed consent.

### 2.2. Methods

The immunohistochemical staining was performed on archive formalin fixed paraffin embedded tissue sections derived from the Department of Clinical Pathomorphology Collegium Medicum, Nicolaus Copernicus University in Torun. The paraffin blocks were cut on 4 *μ*m thick sections and placed on extra adhesive slides (SuperFrostPlus, Thermo Scientific). The proper immunohistochemical staining was followed by a series of positive and negative control reactions. The positive control was performed on model tissue sections, where from reference sources (The Human Protein Atlas) and from manufactured antibodies datasheet, the presence of the analyzed antigens was indicated. The immunohistochemical studies were performed using, respectively, mouse monoclonal antibody against HIF-1*α* (clone [H1ALPHA67], ab1, Abcam, Cambridge, UK), rabbit polyclonal antibody against GLUT-1 (07-1401, MILLOPORE), and rabbit polyclonal antibody against CAIX (NB100-417, NOVUS BIOLOGICALS, Cambridge, UK). The immunohistochemical staining of GLUT-1 (dilution 1 : 200) and CAIX (dilution 1 : 1500) were performed automatically in Dako AurostainerLink48 (Dako, Glostrup, Denmark) and against HIF-1*α* (dilution 1 : 100) was performed manually. Epitopes were unmasked in PT-Link (Dako) using Epitope Retrieval Solution pH-9, subsequently the activity of endogenous peroxidase was blocked by Peroxidase Block (Dako) for 10 minutes, and the nonspecific antibody binding was blocked by 5% BSA (bovine albumin solution) in PBS (Phosphate Buffered Saline). The incubation with primary antibody against GLUT-1 and CAIX was performed for 30 minutes in RT (room temperature) and for HIF-1*α* overnight at 4°C. Furthermore, tissue sections were incubated with EnVision FLEX-HRP (Dako), and the antigen-antibody complex was localized using DAB (3-3′diaminobenzidine) as a chromogen as the brown reaction product.

### 2.3. Examination of Protein Expression

The protein expression was evaluated in light microscope ECLIPSE E800 (Nikon Instruments Europe, Amsterdam, The Netherlands) at 20x original objective magnification. The pathologists who were evaluating the immunohistochemical expression of examined antigens worked independently, and they have been blinded for the patients' clinical as well as other data.

The protein expression was estimated using morphometric principles based on Remmele-Stegner scoring scale [20] [IRS: 0–12; SI x PP], as the ratio of the intensity of protein expression [SI][scale (0–3); 0: negative, 1: low staining, 2: moderate staining, and 3: strong staining] and the percentage of positively stained cells or tissue area [PP] [scale (0–4); 0: negative; 1, <10% positive area; 2, 10–50% positive area; 3, 50–80% positive area; 4, ≥80% positive area].

### 2.4. Statistical Analysis

All statistical analyses were performed using PQStat version 1.4.4.126. The statistical significance of SDF-1, CXCR4, and CXCR7 differences in relation to clinicopathological features was assessed by the use of Kruskal-Wallis and Mann-Whitney *U* test. The overall survival rate was examined for significance using log-rank test and Kaplan-Meier curves. The univariate and multivariate Cox regression were performed. For the analysis, a forward selection with a *P* value of less than 0.05 for entry was applied. The effects of the variables were expressed as hazard ratios per 1 SD change to allow for a better comparability between the effect sizes of the different tested variables. *P* value <0.05 was considered statistically significant.

## 3. Results

**Figure 1:**
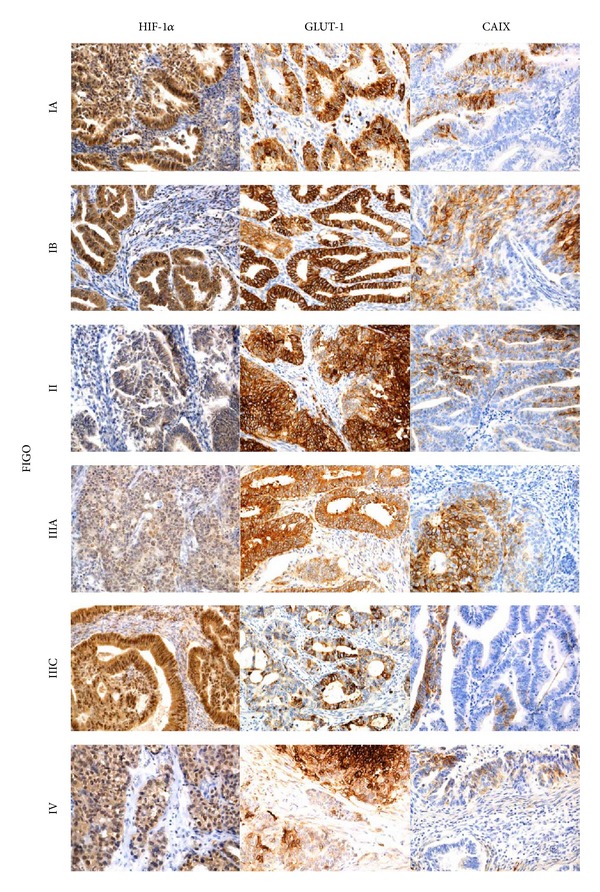
Immunohistochemical representative microphotographs representing the HIF-1*α*, GLUT-1, and CAIX expression in endometrial cancer according to FIGO classification (IA, IB, II, IIIA, IIIC, and IV). Primary objective magnification 20x.

The nuclear expression of HIF-1*α* was found in 97% cases, the cytoplasmic-membranous expression of GLUT-1 was found in 100% cases, and the cytoplasmic-membranous expression of CAIX was found in 89% cases of endometrial cancer (Figure 1).

The results of expression of analyzed proteins according to patients' histological features are shown in Tables 2–4.

Statistical analyses revealed significant differences between the HIF-1*α* expression and Bokhman subtypes of endometrial cancer (*P* = 0.0115).

Moreover, higher HIF-1*α* expression was found for nonendometrial compared to endometrial cancer. High significant difference (*P* = 0.0434) was found between the HIF-1*α* expression and the risk of the recurrence. And higher HIF-1*α*, expression was associated with the higher risk of recurrence (Table 2). However, no statistically significant differences were obtained between the HIF-1*α* expression and the FIGO clinical stage, grading, lymph node, and distant metastases (Table 2). Nevertheless, statistical analyses did not reveal any significant differences between HIF-1*α* expression and deep myometrial invasion (≥50%), cervical involvement, and adnexa involvement (Table 2).

According to GLUT-1 and CAIX expression, the statistical analyses did not reveal any significant differences in these proteins expression and FIGO clinical stage, histological grade (G), the Bokhman subtypes of endometrial cancer, lymph node involvement (N), distant metastases (M), deep myometrial invasion (≥50%), cervical involvement, involvement of adnexa, and recurrence (Tables 3-4).

Moreover, the associations between HIF-1*α*, GLUT-1, and CAIX expression and survival rate was also performed according to univariable and multivariable Cox regression analysis (including variables such as advanced FIGO stage (III + IV), high grade (G3), nonendometrioid subtype (Bokhman II), lymph node metastases, and deep myometrial infiltration (≥50%) (Tables 5-6). Advanced FIGO stage, high grade, lymph node infiltration, and deep myometrial invasion were statistically important prognostic factors in univariate analysis for 5-year survival.

HIF-1*α* expression was conditionally important. Neither nonendometrioid subtype nor GLUT-1 and CAIX expression were important in univariate analysis (Table 5). In multivariate analysis only deep myometrial invasion was statistically important (Table 6).

## 4. Discussion

Hypoxia plays an important role in carcinogenesis. However, despite the well-documented role of hypoxia in tumor microenvironment, its importance in endometrial cancer is not well explained. The growth of tumor requires constant increasing supply of oxygen and nutrients, which enhance development of new vasculature. Frequently, the growth of tumor precedes the development of its blood vessels which is reflected by the presence of hypoxemic areas. These areas are located both in the tumor periphery, as a consequence of enhanced proliferation, and in its central part, where the penetration of blood vessels is insufficient [21]. The hypoxia-induced synthesis of HIF-1*α* causes modification of tumor microenvironment. Moreover, with tumor progression cancer cells become independent of external regulatory factors and can migrate towards body regions which are better supplied in oxygen. It is postulated that the expression of HIF-1*α* is associated with the resistance to chemo- and radiotherapy. Such assumption stimulated research for novel agents that could overcome the therapeutic resistance associated with HIF-1*α* overexpression [22]. It is currently postulated that HIF-1*α* plays a vital role during cellular response to hypoxia. Furthermore, it was revealed that apart from hypoxia, high levels of HIF-1*α* can also result from stimulation with viral oncogenes [23].

It also should be mentioned that HIF-1*α* is involved in endometrial repair during menstrual cycle. Increased expression of HIF-1*α* is associated mostly with the secretory phase, with the peak expression documented during its late stages [24]. Recent studies confirmed the HIF-1*α*-dependent effect of decreased progesterone level and hypoxia on the induction of endometrial secretion of angiogenic factors, interleukin-8 (IL-8), and VEGF [25]. Moreover, HIF-1*α* was revealed to be a coactivator of estrogen-dependent VEGF synthesis [26].

HIF-1*α* modulates angiogenesis not only in normal endometrium but also in endometrial cancer. It was revealed that HIF-1*α*-positive myofibroblasts release VEGF during myometrial invasion [26]. Moreover, hypoxia acts synergistically with prostaglandin E receptor, promoting proliferation of endometrial cancer cells and growth of this tumor in an animal model [27].

HIF-1*α* is involved in the progression of endometrial cancer through the regulation of p27kip cell cycle inhibitor [28]. Moreover, the presence of polymorphism in HIF-1*α* gene is associated with greater stability of HIF-1*α* and its constant activation. The presence of such polymorphism was documented in endometrial cancer cells as a de novo mutation (proline serine 582). This defect was associated with higher vascular density and greater growth potential of the tumor. However, it is unclear if the presence of this polymorphism is associated with increased risk of endometrial cancer as the available data are inconclusive [28, 29].

Moreover, the stabilization of HIF-1*α* can result from activation of PTEN/mTOR signaling pathway. Mutations of the PTEN encoding gene enhance proliferation, increasing expression of cell cycle proteins and DNA replication. In the case of normal endometrial cells this is reflected by their self-regeneration. In contrast, the deactivation of the inhibitory transforming function of PTEN causes malignant transformation of endometrial cells [30]. The protein encoded by the PTEN suppressor gene is a phosphatase responsible for dephosphorylation of cell membrane lipids. Moreover, it acts as an inhibitor of AKT kinase pathway, inhibiting the activity of PI3K. Expression of PTEN protein is documented in approximately 83% of endometrial cancers. Impaired function of PTEN or its lack is reflected by overactivation of PI3K/AKT/mTOR pathway, leading to uncontrolled growth of the tumor. In the case of endometrial cancer cells, the PI3K/AKT/mTOR signal transduction cascade constitutes one of the main activation pathways of tyrosine kinase receptors such as vascular endothelial growth factor receptor 1 (VEGFR-1), platelet-derived growth factor receptor (PDGFR-*α*), epidermal growth factor receptor 1 (EGFR-1), and c-MET [31]. Due to high prevalence of PTEN mutations and resultant activation of PI3K/AKT pathway, stabilization of HIF-1*α* and activation of various target genes are frequently observed in endometrial cancer cells.

Currently it is postulated that the expression of HIF-1*α* increases, from minimal values observed in normal endometrium to intermediate and high levels documented in the case of endometrial hyperplasia and cancer, respectively [28]. The fact that the expression of HIF-1*α* increases proportionally to the clinical stage of the tumor is equally important. The increase in the expression of HIF-1*α* is accompanied by an increase in vascular density, a marker of angiogenesis; this points to the relationship between HIF-1*α*, angiogenesis, and endometrial cancer. Furthermore, the association between the expression of HIF-1*α*, increasing level of angiopoietin-1/angiopoietin-2, and enhanced synthesis of IL-8 was documented, also confirming the role of HIF-1*α* in the angiogenesis of endometrial cancer [32].

Our study confirmed the expression of HIF-1*α* in endometrial cancer. Compared to endometrioid tumors, the expression of anti-HIF-1*α* antibody was significantly higher in nonendometrioid malignancies (*P* = 0.0115). The level of HIF-1*α* was significantly associated with the presence of subtype 2 according to Bokhman. Our findings are consistent with the results of the only previous study analyzing the expression of HIF-1*α* depending on Bokhman's subtype [33]. Pansare et al. revealed that the expression of HIF-1*α* is higher in nonendometrioid type of endometrial cancer. Moreover, these authors showed that the increased expression of HIF-1*α* is associated with the presence of unfavorable prognostic factors (histopathological grade, histological subtype, depth of myometrial invasion, involvement of vascular spaces, and/or adnexa) in patients with Bokhman's subtype 1 of endometrial cancer [33].

Furthermore, we observed that the expression of HIF-1*α* differed significantly depending on the risk of recurrence (*P* = 0.0434). Significantly higher expression of anti-HIF-1*α* antibody was associated with moderate and high risk of recurrence. Both univariate and multivariate analysis of regression revealed that the expression of HIF-1*α* protein is a significant predictor of Bokhman's subtype 2 and is associated with moderate or high risk of recurrence. The results of previous studies on the prognostic value of HIF-1*α* in endometrial cancer are inconclusive and controversial. According to some authors, higher expression of HIF-1*α* is associated with shorter survival and time to recurrence; in contrast, other researchers postulated that HIF-1*α* is not associated with prognosis in endometrial cancer patients [34–37]. Noticeably, our study showed that higher expression of this protein is associated with less favorable type of endometrial cancer (subtype 2) and higher risk of recurrence; this seems consistent with the results of Seeber's and Sivridis' studies [34, 35]. Apart from other reasons, the discrepancies between the results of previous studies may result from the lack of unified standards of HIF-1*α* determination in endometrial cancer patients (solely nuclear versus solely cytoplasmic expression, determination in the whole tumor with or without the necrotic areas) and small size of examined groups. All the facts mentioned above suggest that the role of HIF-1*α* in endometrial cancer prognosis is still not unambiguously explained and further studies of larger groups of patients are needed in order to solve the problem in question.

The idea behind detailed understanding of molecular mechanisms underlying the development of endometrial cancer is to implement targeted therapies improving the outcome of patients with this malignancy. Confirming the involvement of PTEN/mTOR pathway in endometrial carcinogenesis stimulated research on mTOR inhibitors. In a phase 2 study of rapamycin derivative, temsirolimus, partial response was documented in 14% of endometrial cancer patients without previous chemotherapy and in 4% of women with a history of systemic treatment. Furthermore, 69% of previously untreated women and 48% of the patients after chemotherapy showed stabilization of the disease. However, this study did not reveal a relationship between the expression of PTEN protein or the presence of mutation in PTEN gene and the outcome of temsirolimus therapy [38]. Currently other mTOR inhibitors are being tested in the therapy of advanced endometrial cancer.

The expression of HIF-1*α* in a hypoxemic tumor microenvironment changes the metabolism of glucose from aerobic to nonaerobic process. Glucose transporters (GLUTs) and carbonic anhydrases (CAs) are involved in this adaptation to changed aerobic conditions. GLUT-1 is a glucose transporter which is also responsible for the uptake of this sugar. The expression of GLUT-1 increased under nonaerobic conditions, inducing a metabolic shift towards glycolysis. Previous studies failed to document the expression of GLUT-1 in most normal epithelial cells. In contrast, the overexpression of GLUT-1 was confirmed in the case of various neoplasms, for example, in colorectal, esophageal, thyroid, lung, ovarian, and breast cancer [39]. Our study revealed that the expression of GLUT-1 is increased in endometrial cancer as well. However, we did not document significant correlations between the expression of GLUT-1 and clinicopathological characteristics of endometrial cancer. This observation seems consistent with previous reports according to which the presence of GLUT-1 is a marker of neoplastic transformation [40]. The lack of GLUT-1 expression in normal endometrium as well as its weak expression in precancerous lesions and overexpression in endometrial cancer suggests that this molecule can be involved in early stages of endometrial carcinogenesis. According to Xiong et al., the expression of GLUT-1 can be used to distinguish between benign endometrial lesions and endometrial cancer but has no prognostic value in women with this malignancy [39]. This is consistent with the results of our study which did not show significant differences in the expression of GLUT-1 associated with clinical stage or prognosis in endometrial cancer patients.

In order to analyze the hypoxemic tumor microenvironment more comprehensively, we determined its expression of carbonic anhydrase IX (CAIX). This transmembrane HIF-1*α*-dependent glycoprotein is responsible for the regulation of pH in the tumor microenvironment [18]. CAIX plays an important role in the elimination of acids synthesized during the hypoxia-induced glycolysis. The overexpression of CAIX was observed in many neoplasms but not in normal tissues [19]. Our study confirmed increased expression of CAIX in the microenvironment of endometrial cancer. However, we did not observe significant correlations between the level of this expression and clinicopathological characteristics of the tumor. This finding is in line with the results of two studies published to date [41, 42]. Knapp et al. confirmed that the expression of CAIX in endometrial cancer is higher than in normal endometrium and suggested the involvement of anhydrase IX in the shift of glucose metabolism associated with neoplastic transformation [41]. Also Hynninen et al. claimed on the lack CAIX in normal endometrium and its high expression in endometrial cancer tissue [42]. However, the results of our study suggest that the determination of both GLUT1 and CAIX expression is not useful in establishing prognosis in endometrial cancer patients. The involvement of GLUT1 and CAIX in the early stages of carcinogenesis, that is, in the metabolic “shift,” points to their potential application in distinguishing between benign and malignant lesions rather than in prediction of prognosis in endometrial cancer patients.

In conclusion, the important role of HIF-1*α* in the group of patients with the high risk of recurrence and the negative histologic subtype of the tumor suggests that the expression of this factor might be useful in the panel of accessory pathomorphological tests and could be helpful in establishing more accurate prognosis in endometrial cancer patients.

## Figures and Tables

**Table 1:** Baseline characteristics of endometrial cancer patients.

	N (%)
FIGO stage	
IA	27 (29,35%)
IB	18 (19,57%)
II	14 (15,22%)
IIIA	10 (10,87%)
IIIC	17 (18,48%)
IVB	6 (6,52%)
Grading	
G1	7 (7,61%)
G2	66 (71,74%)
G3	19 (20,65%)
Bokhman subtype	
Endometrioid	70 (76,09%)
Nonendometrioid	22 (23,91%)
Lymph node metastases (N)	
Absent N0	69 (75%)
Present N1	23 (25%)
Distant metastases (M)	
Absent M0	86 (93,48%)
Present M1	6 (6,52%)
Myometrial invasion	
<50%	56 (60,87%)
≥50%	36 (39,13%)
Cervical involvement	
Absent	54 (58,70%)
Present	38 (41,30%)
Adnexal Involvement	
Absent	81 (88,04%)
Present	11 (11,96%)

**Table 2:** The HIF1-*α* expression according to clinicopathological features.

	Average	Standard deviation	Minimum	Lower quartile	Median	Upper quartile	Maximum	*P*
FIGO stage								
IA	6.31	3.30	0	4	6	9	12	NS
IB	6.82	2.74	3	4	6	9	12	
II	5.14	1.46	3	4	6	6	8	
IIIA	7.20	2.35	4	6	8	8	12	
IIIC	5.65	3.10	0	4	6	8	12	
IVB	4.33	2.34	0	4	5	6	6	
Grading								
1	5.86	3.48	2	4	4	9	12	NS
2	6.25	2.77	0	4	6	8	12	
3	5.50	2.83	0	4	6	8	9	
Bokhman subtype								
1	5.56	2.29	0	4	6	6	12	0.0115
2	7.64	3.67	0	6	7	12	12	
Lymph node metastases								
0	6.30	2.77	0	4	6	8	12	NS
1	5.39	2.92	0	4	6	6	12	
Distant metastases								
0	6.19	2.82	0	4	6	8	12	NS
1	4.33	2.34	0	4	5	6	6	
Myometrial invasion >50%								
0	6.38	3.07	0	4	6	9	12	NS
1	5.57	2.33	0	4	6	6	12	
Cervical infiltration								
0	6.21	3.07	0	4	6	9	12	NS
1	5.87	2.46	0	4	6	8	12	
Infiltration of adnexa								
0	6.05	2.81	0	4	6	8	12	NS
1	6.18	3.03	0	4	6	8	12	
Risk of recurrence								
Low	4.90	2.15	0	4	5	6	9	0.0434
Intermediate and high	6.40	2.91	0	4	6	8	12	

**Table 3:** The GLUT-1 expression according to clinicopathological features.

	Average	Standard deviation	Minimum	Lower quartile	Median	Upper quartile	Maximum	*P*
FIGO stage								
IA	6.35	2.24	2	6	6	8	12	NS
IB	6.65	1.69	4	6	6	9	9	
II	6.64	1.28	6	6	6	6	9	
IIIA	6.00	0.00	6	6	6	6	6	
IIIC	5.29	1.21	2	4	6	6	6	
IVB	7.50	1.64	6	6	7.5	9	9	
Grading								
1	6.00	2.52	2	4	6	9	9	NS
2	6.31	1.62	2	6	6	6	12	
3	6.33	1.78	2	6	6	6	9	
Bokhman subtype								
1	6.34	1.75	2	6	6	6	12	NS
2	6.14	1.61	2	6	6	6	9	
Lymph node metastases								
0	6.48	1.75	2	6	6	6	12	NS
1	5.74	1.48	2	6	6	6	9	
Distant metastases								
0	6.20	1.69	2	6	6	6	12	NS
1	7.50	1.64	6	6	7.5	9	9	
Myometrial invasion >50%								
0	6.16	1.90	2	6	6	6	12	NS
1	6.49	1.36	4	6	6	6	9	
Cervical infiltration								
0	6.35	2.07	2	6	6	8.5	12	NS
1	6.21	1.07	4	6	6	6	9	
Infiltration of adnexa								
0	6.25	1.77	2	6	6	6	12	NS
1	6.55	1.21	6	6	6	6	9	
Risk of recurrence								
Low	6.20	2.46	2	5	6	7.5	12	NS
Intermediate and high	6.31	1.45	2	6	6	6	9	

**Table 4:** The CAIX expression according to clinicopathological features.

	Average	Standard deviation	Minimum	Lower quartile	Median	Upper quartile	Maximum	*P*
FIGO stage								
IA	3.26	2.61	0	0	3	6	9	NS
IB	4.53	1.74	2	3	6	6	6	
II	4.07	2.16	0	2	5	6	6	
IIIA	4.60	2.55	0	3	5	6	9	
IIIC	4.71	2.52	0	2	6	6	9	
IVB	3.67	2.34	0	2	4	6	6	
Grading								
1	2.86	1.86	0	2	3	4	6	NS
2	4.38	2.39	0	2	6	6	9	
3	3.42	2.29	0	2	3	6	6	
Bokhman subtype								
1	4.19	2.35	0	2	4	6	9	NS
2	3.68	2.46	0	2	4	6	9	
Lymph node metastases								
0	3.96	2.34	0	2	4	6	9	NS
1	4.39	2.48	0	2	4	6	9	
Distant metastases								
0	4.09	2.38	0	2	4	6	9	NS
1	3.67	2.34	0	2	4	6	6	
Myometrial invasion >50%								
0	3.80	2.36	0	2	4	6	9	NS
1	4.49	2.36	0	3	4	6	9	
Cervical infiltration								
0	3.75	2.24	0	2	4	6	9	NS
1	4.50	2.50	0	2	6	6	9	
Infiltration of adnexa								
0	4.04	2.43	0	2	4	6	9	NS
1	4.27	2.00	0	3	4	6	6	
Risk of recurrence								
Low	4.05	2.50	0	2	4	6	9	NS
Intermediate and high	4.07	2.35	0	2	4	6	9	

**Table 5:** Prognostic factors for overall survival selected by Cox's univariate analysis.

	Parameter evaluation	*P* value	HR	HR (95% CI)	
				−95% CI	+95% CI
HIF-1*α*	0.070756	0.092396	0.887741	0.772784	1.019798
GLUT-1	0.112819	0.654167	1.051840	0.843175	1.312143
CAIX	0.079180	0.610033	0.960421	0.822363	1.121656
Figo [III + IV]	0.196299	0.002108	0.299063	0.138543	0.645570
G3	0.204699	0.026614	0.403454	0.180847	0.900066
Bokhman's subtype 2	0.210773	0.606716	0.804931	0.352320	1.838992
N+	0.193138	0.001314	0.289078	0.135586	0.616332
Mm [>50%]	0.200038	0.000652	0.255676	0.116720	0.560059

CI: confidence interval; FIGO: Federation Internationale de Gynecologie et d'Obstetrique; HR: hazard ratio; N+: lymph node involvement.

**Table 6:** Prognostic factors for overall survival selected by Cox's multivariate analysis.

	Parameter evaluation	*P* value	HR	HR (95% CI)	
				−95% CI	+95% CI
HIF-1*α*	0.071684	0.406520	0.942235	0.818732	1.084369
GLUT-1	0.131077	0.642657	1.062705	0.821940	1.373996
CAIX	0.085198	0.545842	0.949842	0.803769	1.122462
Figo [III + IV]	0.400904	0.868605	0.875777	0.181925	4.215931
G3	0.246020	0.448415	0.688668	0.262533	1.806490
Bokhman's subtype 2	0.223343	0.678850	0.831152	0.346307	1.994802
N+	0.336866	0.436507	0.592009	0.158069	2.217227
Mm [>50%]	0.278537	0.043148	0.340540	0.114284	1.014729

CI: confidence interval; FIGO: Federation Internationale de Gynecologie et d'Obstetrique; HR: hazard ratio; N+: lymph node involvement.
